# There’s an App for That!

**Published:** 2014-01-01

**Authors:** Wendy H. Vogel

What would we do without our smartphones? There are so many applications, or "apps," and so little time! In a long-awaited report released in 2012, Strategy Analytics estimated that there are more than 1 billion smartphone users worldwide (Bicheno, 2012). It is even more relevant to note that an estimated 70% to 85% of health-care providers in the United States use smartphones (Ozdalga, Ozdalga, & Ahuja, 2012). With apps created specifically with the busy clinician in mind, many people feel that having quickly accessible information improves care at the bedside.

## A Cautionary Note

For advanced practitioners (APs), it is critical to keep smartphone use in perspective and never take for granted the best source of information: face-to-face contact with the patient. According to a 2011 The New York Times article, the "distracted doctoring" phenomenon is a true concern (Richtel, 2011). So with the ever-growing use of smartphones and apps meant for the clinician, we need to remember to ask a key question: Are we spending too much time looking at our phones and not enough time looking at our patients? Balanced use of available technology is a win-win for both the advanced practitioner and the patient.

## Medical Apps for Patients

More and more often, patients are turning to apps that can help them find reliable medical information, track details regarding their health, and even stay on a course of beneficial lifestyle changes, all with the goals of improved health and overall well-being.

In Tables 1 and 2 on the next page, you’ll find a collection of some of this author’s "finds" of the month. Table 1 consists of apps that may be of interest to the advanced practitioner, while Table 2 is a collection of apps intended for patient use. Take a look through this list and see which of these tools appeal to you.

**Table 1:**
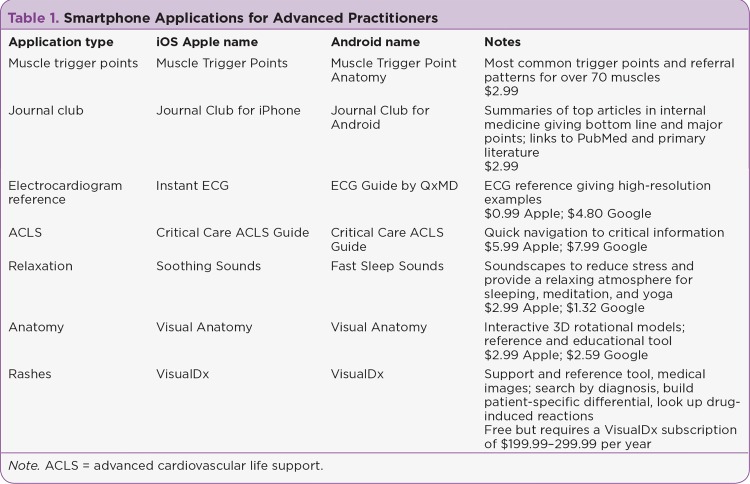
Table 1. Smartphone Applications for Advanced Practitioners

**Table 2:**
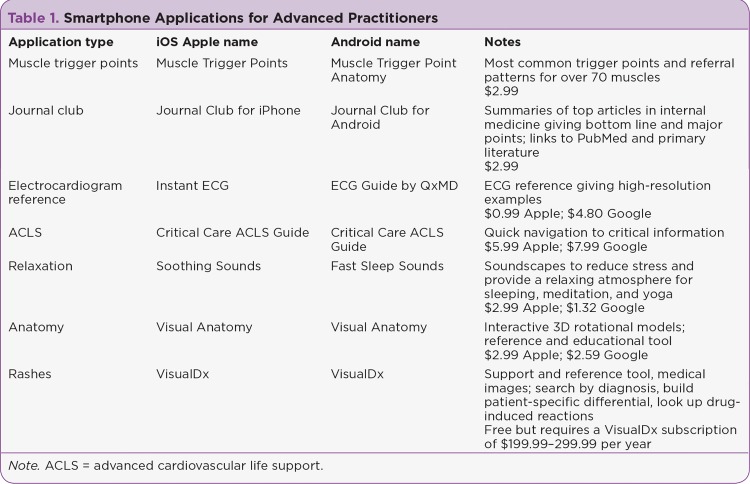
Table 2. Smartphone Applications for Patients
